# Effects of agricultural management on phyllosphere fungal diversity in vineyards and the association with adjacent native forests

**DOI:** 10.7717/peerj.5715

**Published:** 2018-10-29

**Authors:** Luis E. Castañeda, Toshiko Miura, Roland Sánchez, Olga Barbosa

**Affiliations:** 1 Programa de Genética Humana, Instituto de Ciencias Biomédicas, Facultad de Medicina, Universidad de Chile, Santiago, Chile; 2 Instituto de Ciencias Ambientales y Evolutivas, Facultad de Ciencias, Universidad Austral de Chile, Valdivia, Chile; 3 Research Institute of Environment, Agriculture and Fisheries, Osaka Prefecture, Japan; 4 Instituto de Ecología y Biodiversidad, Santiago, Chile

**Keywords:** Amplicon sequencing, Chile, Ecosystem services, Organic management, Yeast, Mediterranean biome

## Abstract

Agriculture is one of the main drivers of land conversion, and agriculture practices can impact on microbial diversity. Here we characterized the phyllosphere fungal diversity associated with Carménère grapevines under conventional and organic agricultural management. We also explored the fungal diversity present in the adjacent sclerophyllous forests to explore the potential role of native forest on vineyard phyllosphere. After conducting D2 and ITS2 amplicon sequencing, we found that fungal diversity indices did not change between conventional and organic vineyards, but community structure was sensitive to the agricultural management. On the other hand, we found a high proportion of shared fungal OTUs between vineyards and native forests. In addition, both habitats had similar levels of fungal diversity despite forest samples were derived from multiple plant species. In contrast, the community structure was different in both habitats. Interestingly, the native forest had more unidentified species and unique OTUs than vineyards. Forest dominant species were *Aureobasidium pullulans* and *Endoconidioma populi*, whereas *Davidiella tassiana*, *Didymella sp.*, and *Alternaria eichhorniae* were more abundant in vineyards. Overall, this study argues that a better understanding of the relationship native forests and agroecosystems is needed for maintaining and enhancing ecosystem services provided by natural ecosystems. Finally, knowledge of microbial communities living in the Chilean Mediterranean biome is needed for appropriate conservation management of these biomes and their classification as biodiversity hotspots.

## Introduction

Land conversion is one of the most important drivers of habitat loss, changing the biophysical conditions of natural ecosystems and affecting the ecosystem functioning of these habitats (Vitousek et al., 1997; Griffiths & Philippot, 2013). One of the main drivers of land conversion is agriculture, which has transformed forests and grasslands into arable land for food production (Fiedler, Landis & Wratten, 2008). Agriculture involves management practices and the addition of chemical or organic products to improve growth plant, increase plant biomass, eliminate crop pests, and reduce weed competence (Fiedler, Landis & Wratten, 2008). These practices can be classified into two main categories: conventional or organic management (Coll et al., 2011). Conventional agriculture involves the application of inorganic fertilizer (e.g., nitrogen and phosphorous) to improve plant growth, and synthetic insecticides and herbicides are used to control weed competition. Conversely, organic agriculture employs organic fertilizers (e.g., compost, humus), biological control to manage pests, and tillage or grass-cutting to manage weeds.

Habitat conversion and agricultural management have profound effects on the physical and biological properties of agroecosystems. For example, water and soil quality, microbial community structure, invertebrate abundance, and bird species richness have all been shown to be affected by agriculture management and habitat conversion (Coll et al., 2011; García-Orenes et al., 2013). In light of this, organic farming has been proposed as a potential agricultural practice to increase biodiversity in farmlands (Hole et al., 2005; Chamberlain et al., 2010). On the other hand, Gabriel et al. (2010) report that organic farms have positive effects on the diversity of plants, bumblebees, and butterflies, but not necessarily on hoverflies and birds. Agricultural practices can also affect the diversity of microorganisms in agroecosystems such as vineyards (Bevivino et al., 2014; Castañeda et al., 2015; García-Orenes et al., 2013). For instance, the application of organic matter (e.g., oat straw) increases fungal abundance in managed soils in vineyards and results in a microbial community structure similar to that found in forest soils (García-Orenes et al., 2013). Agricultural management can also influence the bacterial microbiota associated with the surfaces of leaves, fruits, and vegetables, otherwise known as the phyllosphere (i.e., the microbial habitat found on the above-ground surface of plants) (Ottesen et al., 2013). Indeed, Karlsson et al. (2014) have found significant effects of fungicide use on the fungal evenness of wheat phyllosphere, but the fungicide use had no effect on wheat fungal pathogens. The effect of vineyard management on fungal soil community structure has also been reported, where organic and biodynamic vineyards support higher fungal diversity than conventional vineyards (Setati et al., 2012; Bagheri, Bauer & Setati, 2015). However, the effects of agricultural practices on phyllosphere microbiota have only recently been studied, and it is yet not well known how the presence and abundance of key microorganisms affect food or wine production (but see Perazzolli et al., 2014; Morrison-Whittle, Lee & Goddard, 2017).

Winemaking relies on the microbial contribution of bacteria and yeasts from grapevine growth to wine fermentation (Fleet, 2008; Mills et al., 2008). In particular, yeasts play important roles in alcoholic fermentation, consuming sugar and producing ethanol but also contribute to the sensorial features of wine (Fleet, 2008; Renouf et al., 2006). Most wineries around the world employ commercial yeasts (e.g., *Saccharomyces cerevisiae*) to control fermentation, but recent evidence indicates that grape microbiota plays an important role in both spontaneous and inoculated fermentation, contributing to the taste and flavor of wine (Ganga & Martinez, 2004; Renouf et al., 2006). Recently, it has been shown that the diversity of grape microbiota is associated with local environmental conditions, suggesting that microbial *terroir* does influencing the organoleptic features of wine (Bokulich et al., 2014; Bokulich et al., 2016).

Reduced habitat heterogeneity as a consequence of habitat conversion could also influence the fungal diversity of vineyards. It has been reported that plant diversity is linked to soil fungal diversity because a higher number of plant hosts increases the availability of potential fungi-host interactions (Chung et al., 2007; Holland et al., 2016). However, despite that some studies have explored the differences in soil microbiota between vineyards and surrounding vegetation (Orgiazzi et al., 2012; Holland et al., 2016), there is little knowledge about how the phyllosphere differs between managed and unmanaged habitats. Furthermore, native vegetation has been demonstrated to be a suitable habitat for fermenting yeasts (Kurtzman, Fell & Boekhout, 2011). For example, *Saccharomyces* fungi have been found growing on soil and on tree surface (Libkind et al., 2011; Sampaio & Gonçalves, 2008). Additionally, fermenting yeasts can be found in honeydew, a sugary fluid excreted by aphids feeding on trees (Serjeant et al., 2008). Therefore, the high availability of substrates and hosts in native forests compared to vineyards should lead to increased fungal diversity in forests.

In the present study, we characterized the fungal diversity of Carménère vines subjected to different agricultural practices (conventional and organic management). For this goal, we employed an amplicon sequencing approach to explore the fungal species composition and community diversity in six organic and conventional vineyards in central Chile. Particularly, we focused on Carménère vines because they suffered a phylloxera infestation in 1867 in Europe that greatly reduced the area cultivated with this vine, and currently the largest Carménère cultivar is found in Chile (Moncada & Hinrichsen, 2007). However, despite its historic and enological value, Carménère microbiota has not been deeply studied except for a recent work of Miura et al. (2017). They explored the geographical patterns in bacterial (16S rRNA sequences) and fungal (ITS2 sequences) microbiota associated with Carménère to test a diversity-distance relationship in this agroecosystem. In the present study, we used the ITS2 data employed by Miura et al. (2017), and additionally we sequenced the D2 amplicon in the same samples. We also studied the fungal diversity associated with the phyllosphere of Chilean sclerophyllous trees, sequencing the D2 and ITS2 amplicons. Sclerophyllous forest ecosystem is considered as a biodiversity hotspot because it harbors high plant endemism but it is also threatened by diverse human activities; thus, it is a priority for conservation management (Myers et al., 2000). However, knowledge of the fungal communities of this biome is scarce, and studies employing genomics could provide valuable information for conservation strategies in these areas (Heilmann-Clausen et al., 2015)

## Materials and Methods

### Sampling

Samples were collected during 2014 from six vineyards (three conventional vineyards and three organic vineyards) and surrounding sclerophyllous forest. All vineyards and forests are located in the Colchagua Valley, Chile (34°15′S–34°50′S; 70°15′W–72°00′W), and samples were taken during the last week before the Carménère harvest (April in the Southern Hemisphere).

In each vineyard, three plots containing Carménère cultivars and located close to sclerophyllous forest were chosen. Within each plot, undamaged grape berries and leaves were collected from three vines located close to the border with the forest and from three vines located 30 m toward the center of the vineyard plot. In the forest, leaves were collected from four trees at the border with the vineyard and from four trees located 30 m toward the center of the forest plot. If at the sampling point there were more than one tree species, the sample was composed equally of all species. Common native tree species in the Chilean sclerophyllous forest were litre (*Lithrea caustica*), boldo (*Peumus boldus*), peumo (*Cryptocarya alba*), quillay (*Quillaja saponaria*) and espino (*Acacia caven*). Fruits from forest trees were almost absent during the autumn, and they were not included in the sampling. All of the samples were collected using surgical gloves and sterilized scissors. Upon collection, the samples were stored in sterilized hermetic plastic bags and maintained on dry ice until arrival at the laboratory at the Universidad Austral de Chile (Valdivia, Chile). At the laboratory, the samples were stored at −20 °C until DNA extraction.

### DNA extraction, PCR and amplicon sequencing

Each grape berry sample consisted of 44 grapes, which was divided into four groups with an equal number of grapes and transferred to four 50 ml tubes containing 30 ml of a 0.9% NaCl–0.02% Tween20 solution (therefore washing solution). For each leaf samples (vine or native tree leaves), we took 20 g of leaves, which was divided into four groups with the same quantity of leaves and transferred to four 50 ml tubes containing 30 ml of washing solution. Tubes containing plant material were shaken for 2 h at 100 rpm in a RS-60 multirotator (BioSan, Latvia) at room temperature. The wash solutions were filtered using sterilized gauze to eliminated large pieces of plant tissue. Then, the solutions were centrifuged for 5 min at 1,500 rpm. The supernatant was transferred to new 50 ml tubes and centrifuged for 20 min at 7,500 rpm. Genomic DNA was extracted from the resulting pellets using a PowerSoil DNA isolation kit (MoBio Laboratories, Carlsbad, CA, USA) following the manufacturer’s instructions. After extraction, DNA was quantified employing a fluorescence method with a Quan-iT PicoGreen dsDNA kit (Invitrogen, United States). All samples (grape berries, vine leaves, and native tree leaves) were processed following the same protocol. DNA was extracted from individual plants but then pooled into a single sample for each plot to reduce sequencing costs. Thus, our final sample size was (6 vineyards × 3 plots): 18 grape berry samples, 18 vine leaf samples, and 18 native tree leaf samples.

We amplified two genomic regions (ITS2 and D2/LSU) to characterize fungal diversity. Specifically, we chose the following primers according to Pinto et al. (2014): ITS2-F (5′- GCATCGATGAAGAACGC-3′) and ITS2-R (5′-CCTCCGCTTATTGATATGC-3′); and D2-F (5′-AAGMACTTTGRAAAGAGAG-3′) and D2-R (5′-GGTCCGTGTTTCAAGACG-3′). These pairs of primers have been described as complementary for the identification of fungi associated with vineyards (Pinto et al., 2014). We used a two-step PCR amplicon sequencing. First, an amplicon PCR was performed to amplify each one of the specific molecular markers. The amplicon PCR mix had a final volume of 25 µl: 12.5 µl 2×  KAPA HiFi HotStart ReadyMix (KAPA Biosystems, Wilmington, MA, USA); 5 µl 1 µM forward primer (D2 or ITS2); 5 µl 1 µM reverse primer (D2 or ITS2); and 2.5 µl template DNA (3 ng/µl). PCR cycle conditions were set up as follows: denaturation at 95 °C for 3 min; 25 amplification cycles of 95 °C for 30 s, 55 °C for 30 s and 72 °C for 30 s; and a final extension at 72 °C for 5 min. PCR amplicons were loaded in an agarose gel to check PCR amplification and then, PCR amplicons were purified using AMPure XP beads (Beckman Coulter Inc., Indianapolis, IN, USA). Second, the index PCR was performed to attach Nextera XT DNA indexes (Illumina Corporation, San Diego, CA, USA). The PCR mix had a final volume of 45 µl: 5 µl PCR amplicon, 5 µl Nextera XT DNA index primer i5; 5 µl Nextera XT DNA index primer i7; 25 µl 2×  HiFi HotStart ReadyMix (KAPA Biosystems, Wilmington, MA, USA); and 10 µl nuclease-free water. PCR cycle conditions were set up as follows: denaturation at 95 °C for 3 min; 8 amplification cycles of 95 °C for 30 s, 55 °C for 30 s and 72 °C for 30 s; and a final extension at 72 °C for 5 min. Index PCR products were loaded in a 2% agarose gel and purified using the MiniElute PCR Purification kit (Qiagen, Germantown, MA, USA). Purified PCR products were quantified by fluorescence using the Quant-iT PicoGreen kit (Invitrogen, Grand Island, NY, USA) and sequenced in an Illumina MiSeq sequencer using a 250-bp MiSeq Reagent Kit according to Australomics protocols (Australomics Core Facility, Universidad Austral de Chile).

### Data analysis

Quality of sequences was checked using FastQC (Andrews, 2010). Then, raw sequences were quality filtered for a *Q*-value higher than 26 and for sequences longer than 150 bp using the script Reads_Quality_Length_distribution.pl (Balint et al., 2014). Forward and reverse filtered sequences were paired using Pandaseq with a minimum overlap of 5 bp (Masella et al., 2012). After this, each paired-end sequence file was split into two different files using Fqgrep (https://github.com/indraniel/fqgrep) with each file only contained sequences starting with the ITS or D2 primer sequences. Overall, a total of 108 fastq files were produced (54 samples × 2 amplicons). These files were then converted into fasta files, merged into one single file, and primer sequences were trimmed.

Quality-filtered and trimmed sequences were analyzed using QIIME v1.9.1 (Caporaso et al., 2010a). Operation taxonomic units (OTUs) were clustered with the pick-open-reference-otus script at 97% identity level (threshold value according to Pinto et al., 2014; Matulich et al., 2015; Pinto et al., 2015) using uclust with a percentage of failure sequences of 10% (Edgar, 2010). The taxonomic assignment of OTUs picked from the ITS reads was performed using BLAST against the UNITE fungal database version 7 (Abarenkov et al., 2010). The assignment of OTUs picked from the D2 reads was performed using BLAST against the 28S LSU RDP database version 7 (Liu et al., 2012). In contrast to ITS, the D2/LSU region is suitable for phylogenetic analysis because this molecular marker contains conserved regions, which can be aligned (Porter & Golding, 2012; Lindahl et al., 2013). D2 sequences were aligned against a template alignment from the 28S LSU RDP database using PyNAST (Caporaso et al., 2010b). Then, a phylogenetic tree was constructed with the aligned sequences using FastTree (Price, Dehal & Arkin, 2009). For both amplicons, we removed OTUs matching non-fungal sequences and with total abundances less than 0.001% in the final OTU table.

All downstream analyses were performed in R using the DESeq2 (Love, Huber & Anders, 2014), lme4 (Bates, Maechler & Bolker, 2011), phyloseq (McMurdie & Holmes, 2013), and vegan packages (Oksanen et al., 2013). First, samples were rarefied to the sample with the lowest number of sequences to standardize the number of sequences among samples. Then, we estimated OTU richness, Shannon diversity, and Pielou evenness for the D2 and ITS2 amplicon dataset. We also estimated the phylogenetic diversity (Faith’s PD) using the D2 amplicon. Before the statistical analyses, we checked the normality and the presence of outliers in the dataset. We consistently found an outlier sample (a grape berry sample collected from a conventional vineyard) with extremely lower diversity indices. Preliminary analysis showed significant differences for diversity indices compared between vineyards with different managements and the removal of this single outlier resulted in non-difference between vineyards. Then, this outlier was removed from the dataset to avoid spurious effects associated with the agricultural managements applied to vineyards. A linear mixed model was performed to analyze the diversity indices with agricultural management (conventional and organic management) and plant tissue (grape berries and vine leaves) as fixed effects, and vineyards nested within agricultural management as a random effect. Also, a linear mixed model with habitat as fixed effect (vine leaves and forest leaves) and vineyard as nested within habitat as random effect to compare diversity indices between habitats. The effect of the random effect was evaluated using a likelihood-ratio test comparing the complete model (with the random effect) and the reduced model (without the random effect). We compared the fungal community structure between the agriculture management and plant tissue conducting a two-way PERMANOVA using the adonis function of the vegan R package (Oksanen et al., 2013). We also performed a one-way PERMANOVA to compare the community structure between vineyards and native forests. The PERMANOVA included vineyards as a random effect to account for variability among vineyards. We also removed the ‘outlier’ sample detected in the diversity index analyses from both matrices. PERMANOVA was based on the Bray-Curtis distance matrix for the ITS2 amplicon and the normalized weighted-Unifrac matrix for the D2 amplicon (Lozupone & Knight, 2005). These distance matrices were also used to visualize the fungal community structure in non-metric dimensional scaling (NMDS) plots.

Finally, we compared the OTU abundances across categorical effects (agricultural management, vine tissue, and habitat) using a Wald test in DESeq2 and *P* values were corrected by the Benjamini–Hochberg procedure for multiple comparisons to control for false discovery rate (FDR). We also used the ITS2 OTU table to compared the species abundances across categorical effects (agricultural management, vine tissue, and habitat) using an ANOVA performed in QIIME and *P* values were corrected for FDR. This comparison was based on the species representation within each sample obtained with the summary_taxa script of QIIME. We performed separated analyses for OTU and species abundance because different OTUs can be assigned to the same species. We also characterized the core microbiome of each sample using QIIME and the resulting OTU lists were used to construct Venn diagrams and visualize the number of exclusive and shared OTUs between different types of samples. The Venn diagrams were plotted with Venny 2.1 (Oliveros, 2007).

## Results

For ITS2, a total of 3,606,629 raw sequences were analyzed with the QIIME pipeline. After processing with QIIME, we obtained 3,567,599 sequences that clustered into 897 fungal OTUs (97% sequence similarity), and the samples were rarified at 19,450 sequences (Fig. S1A). For D2, a total of 4,135,846 raw sequences were analyzed with the QIIME pipeline. After processing with QIIME, 4,115,319 sequences clustered into 615 fungal OTUs (97% sequence similarity), and the samples were rarified at 26,350 sequences (Figs. S1B–S1C).

### Fungal diversity within vineyards: management and plant tissue effects

**Figure 1:**
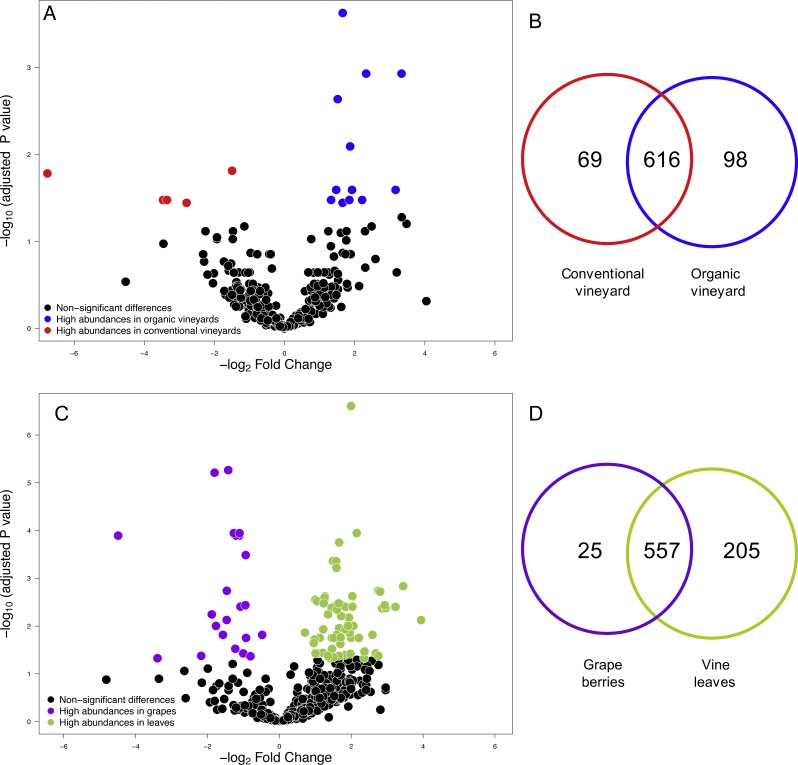
Fungal operational taxonomic units (OTUs) associated with agricultural management and plant tissue in Carménère vineyards. (A) Volcano plot showing OTUs that were significantly more abundant in conventional vineyards (red circles: *P* < 0.05 after FDR correction) and OTUs that were significantly more abundant in organic vineyards (blue circles: *P* < 0.05 after FDR correction). (B) Venn diagram showing OTUs exclusive to conventional (red) and organic (blue) vineyards and OTUs shared between vineyards with different agricultural management. (C) Volcano plot showing OTUs that were significantly more abundant in grape berries (purple circles: *P* < 0.05 after FDR correction) and OTUs that were significantly more abundant in vine leaves (green circles: *P* < 0.05 after FDR correction). For A and C: black circles represent OTUs that did not significantly differ between agricultural management and vine tissues, respectively; each point represents an individual OTU, the *x*-axis indicates the fold change of abundance, and the *y*-axis indicates the adjusted *P* values after FDR correction. (D) Venn diagram showing OTUs exclusive to grape berries (purple) and vine leaves (green) and OTUs shared between plant tissues. Venn plots exclude OTUs with abundances less than 0.01%.

We compared the relative abundances of 897 fungal OTUs between agricultural management (organic and conventional). From this, the relative abundances of only 18 fungal OTUs differed between management types (FDR: *P* < 0.05; Table S1). Of these 18 fungal OTUs, 5 OTUs were more abundant in conventional vineyards and 13 OTUs were significantly more abundant in organic vineyards (Fig. 1A). From the 616 OTUs (78.7%) found in 90% of the samples, only 30 OTUs were found in all vineyard samples (the core vineyard microbiome). Furthermore, 69 OTUs (8.8%) were exclusively found in conventional vineyards, and 98 OTUs (12.5%) were only found in organic vineyards (Fig. 1B). Regarding taxon abundance, we found that only the relative abundance of the *Dothioraceae* family was significantly different between types of agricultural management (conventional = 3.9%; organic = 10.5%; FDR: *P* = 0.02; Table S2). Within this family, the most abundant species was *Aureobasidium pullulans* and despite that it showed higher abundances in organic (9.9%) than in conventional vineyards (3.6%), these differences were not significant after corrections for multiple comparisons (FDR: *P* = 0.092).

**Figure 2:**
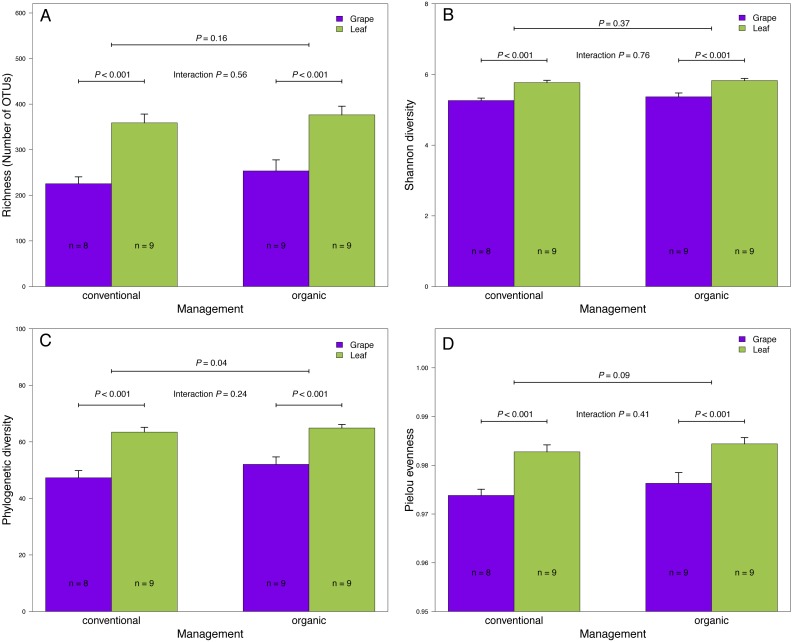
Fungal diversity indices associated with agricultural management and plant tissue in Carménère vineyards. (A) Richness (number of OTUs), (B) Shannon diversity, (C) Phylogenetic diversity, and (D) Pielou evenness estimated for fungal communities sampled in grape berries and vine leaves from conventional and organic vineyards. *P* values were estimated using mixed-linear models with agricultural management and plant tissue as fixed effects and vineyards as random effect. Colored bars represent the mean values for each category, bar errors represent the standard errors, and ‘n’ represents the sample size of each group.

We also compared the relative abundances of fungal OTUs associated with grape berries and vine leaves (Fig. 1C), and we found that the abundances of 22 OTUs were more abundant in grape berries, while 65 OTUs were significantly higher in vine leaves (FDR: *P* < 0.05; Table S3). Shared OTUs between grape berries and vine leaves were 557 (70.8%), whereas 25 OTUs (3.2%) were only found in grape berries and 205 OTUs (26%) were only found in vine leaves (Fig. 1D). Comparing species abundances, we found that 55 species showed significant different abundance between plant tissues (after FDR correction). *Davidiella tassiana* was the most abundant species within vineyards and it was relatively more abundant in grape berries than in vine leaves (FDR: *P* = 0.007; Table S4).

**Figure 3:**
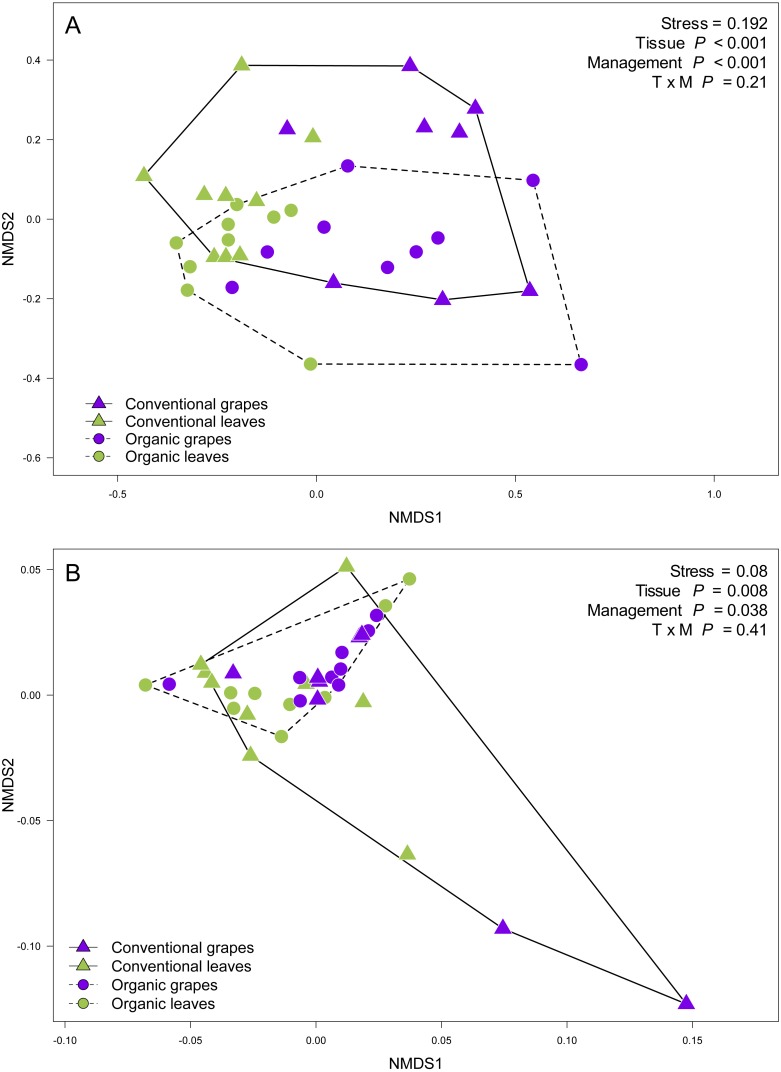
Fungal community structure associated with agricultural management and plant tissue in Carménère vineyards. Non-metric multidimensional scaling (NMDS) plot of fungal communities sampled from grape berries (purple circles) and vine leaves (green circles) of organic vineyards (dashed line), and from grape berries (purple triangle) and vine leaves (green triangle) of conventional vineyards (solid line). (A) ITS2 and (B) D2 amplicon sequences were used to characterize fungal communities. *P* values for plant tissue, agricultural management, and interaction (T × M) effects were estimated using PERMANOVA.

We found that fungal OTU richness, Shannon diversity, phylogenetic diversity, and Pielou evenness were significantly higher in vine leaves than grape berries (Fig. 2, Table 1). On the other hand, these diversity indices were not significantly different between agricultural management, showed non-significant interactions between plant tissue and management, and vineyards did not add a significant variation for any diversity indices (Fig. 2; Table 1). These findings were consistent for both D2 and ITS2 amplicons used in this study (Table 1). Regarding the fungal community structure estimated using ITS2, we found significant effects because of plant tissue (PERMANOVA: *F*_1,31_ = 4.33, *P* < 0.001) and agricultural management (PERMANOVA: *F*_1,31_ = 1.74, *P* < 0.001), but non-significant interaction was detected (PERMANOVA: *F*_1,31_ = 1.07, *P* = 0.21). These differences can be clearly visualized in the NMDS plot, where we can see that vine leaves and grape berries are separated along the NMDS1 axis (green and purple symbols, respectively Figure 3A), while conventional and organic vineyards are separated along the NMDS2 axis (triangle and circle symbols, respectively Figure 3A). We found similar result for the D2 amplicon: a significant effect of plant tissue on the community structure (PERMANOVA: *F*_1,31_ = 4.01, *P* = 0.008); a significant effect of agricultural management (PERMANOVA: *F*_1,31_ = 2.111, *P* = 0.038); and a non-significant interaction between main effects (PERMANOVA: *F*_1,31_ = 0.97, *P* = 0.41). Differences between vine tissues or agricultural management can be explained because fungal communities sampled from grape berries and conventional vineyards show a higher variability than those sampled from leaves or organic vineyards (Fig. 3B).

### Fungal diversity comparison between vineyards and native forest

**Figure 4:**
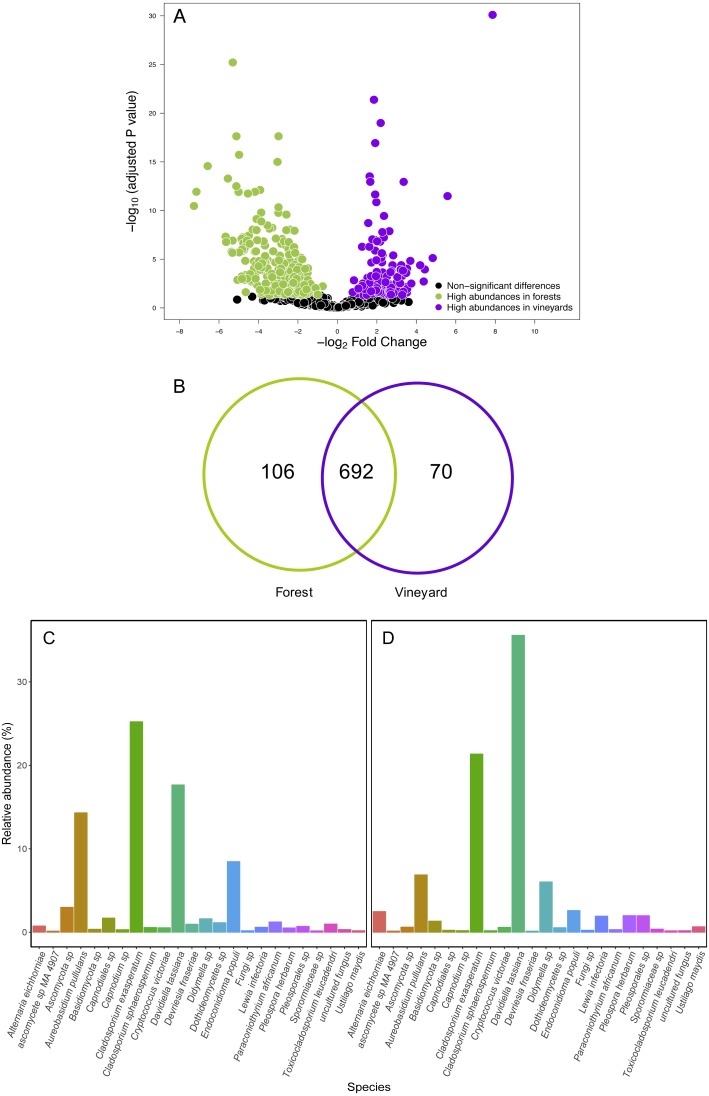
Fungal operational taxonomic units (OTUs) associated with Carménère vineyards and sclerophyllous native trees. (A) Volcano plot showing OTUs that are significantly more abundant in leaves sampled from native trees (green circles: *P* < 0.05 after FDR correction) and OTUs that were significantly more abundant in leaves sampled from grapevines (purple circles: *P* < 0.05 after FDR correction). Black circles represent OTUs that were non-significantly more abundant in either habitat. Each point represents an individual OTU, the *x*-axis indicates the fold change of abundance, and the *y*-axis indicates the adjusted *P* values after FDR correction. (B) Venn diagram showing exclusive OTUs found in forest (green) and vineyard (purple) samples, and OTUs found in both habitats. (C) Relative abundances of fungal species found in forest and vineyard (D) samples. These plots contain the 95% of OTUs found in at least two samples.

We compared OTU relative abundances between forest leaves and grape leaves and we found 351 OTUs that differed in terms of their relative abundances (FDR: *P* < 0.05; Table S5). Of these, 230 fungal OTUs were significantly more abundant in forests, and 124 fungal OTUs were more abundant in vineyards (Fig. 4A). We also explored the presence of OTUs found in at least 90% of the samples: 692 OTUs (79.7%) were present both in forest and vineyard samples, but only 44 OTUs were found in all of the 54 samples representing the core microbiome in this agroecosystem. Additionally, 106 OTUs (12.2%) were exclusively found in the forest samples, and 70 OTUs (8.1%) were only found in vineyard samples (Fig. 4B). Fungal OTUs found in the present study were classified into 432 species, and the most abundant were *Davidiella tassiana* (vineyards = 42.3%; forest = 17.7%; FDR: *P* < 0.0001), *Cladosporium exasperatum* (vineyards = 22.2%; forest = 25.2%; FDR: *P* = 0.26), *Aureobasidium pullulans* (vineyards = 6.7%; forest = 14.3%; FDR: *P* = 0.007), *Endoconidioma populi* (vineyards = 1.9%; forest = 8.5%; FDR: *P* < 0.0001), *Didymella sp.* (vineyards = 4.5%; forest = 1.6%; FDR: *P* = 0.0001). In total, we found that the abundances of 135 species were significantly different after FDR correction for multiple comparisons. Of these, the relative abundances of 114 species were significantly higher in forest samples, and 21 species were more abundant in vineyard samples (Figs. 4C and 4D; Table S6). We also searched for yeasts involved in winemaking (i.e., Saccharomycetales), but the relative abundances of these yeasts were extremely low in all samples (<0.01%). Yeasts belonging to the genera *Hanseniaspora* and *Saccharomyces* were more abundant in vineyards than in forest samples. Despite this, these differences in abundances were not significant between habitats with the exception of *Metschnikowia* that had a significantly higher relative abundance in the vineyard samples (FDR: *P* < 0.0001) (Table S6).

**Table 1:** Fungal diversity indices associated with agricultural management and plant tissue in Carménére vineyards. Results of linear mixed models for the diversity indices estimated from fungal communities sampled from grape berries and vine leaves in conventional and organic vineyards. The *t* values, degrees of freedom and *P* values for fixed effects (vine tissue, agricultural management and its interaction) are obtained from the lmer summary. *P* values of the random effect (vineyard) are based on likelihood-ratio tests (*χ*^2^).

**Diversity index**	**Amplicon**	**Tissue**	**Management**	**Interaction**	**Vineyard**
OTU richness	ITS2	*t*_32_ = 5.07(*P* < 0.001)	*t*_32_ = 1.43 (*P* = 0.16)	*t*_32_ = − 0.58 (*P* = 0.56)	${\chi }_{1}^{2}=0$ (*P* = 1)
	D2	*t*_27_ = 5.86(*P* < 0.001)	*t*_12_ = 1.23 (*P* = 0.24)	*t*_27_ = − 0.44(*P* = 0.67)	${\chi }_{1}^{2}=0 (P=1)$
Shannon diversity	ITS2	*t*_27_ = 4.52(*P* < 0.001)	*t*_13_ = 0.93 (*P* = 0.36)	*t*_27_ = − 0.31(*P* = 0.76)	${\chi }_{1}^{2}=0$ (*P* = 1)
	D2	*t*_27_ = 6.04(*P* < 0.001)	*t*_11_ = 1.34(*P* = 0.21)	*t*_27_ = − 0.62(*P* = 0.54)	${\chi }_{1}^{2}=0.01$ (*P* = 0.91)
Phylogenetic diversity	D2	*t*_27_ = 5.52(*P* < 0.001)	*t*_10_ = 1.39(*P* = 0.20)	*t*_27_ = − 0.77(*P* = 0.45)	${\chi }_{1}^{2}=0.01$ (*P* = 0.90)
Pielou evenness	ITS2	*t*_28_ = 3.89(*P* < 0.001)	*t*_13_ = 1.03(*P* = 0.32)	*t*_27_ = − 0.26(*P* = 0.80)	${\chi }_{1}^{2}=0$ (*P* = 1)
	D2	*t*_28_ = 4.70(*P* < 0.001)	*t*_14_ = 1.55(*P* = 0.14)	*t*_27_ = − 0.73(*P* = 0.47)	${\chi }_{1}^{2}=0$ (*P* = 1)

**Figure 5:**
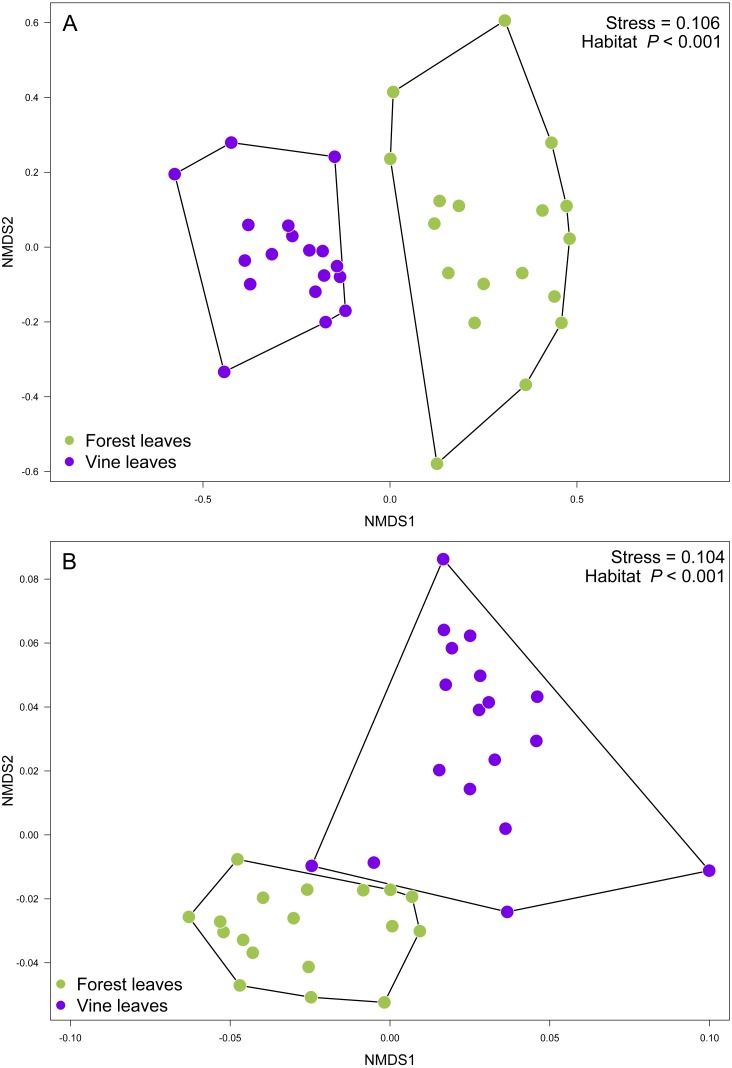
Fungal community structure associated found in Carménère vineyard and native forests. Non-metric multidimensional scaling (NMDS) plot of fungal communities inhabiting forests (green circles) and vineyards (purple circles). Fungal community structure was assessed using (A) ITS2 and (B) D2 amplicons. *P* values for habitat effect were estimated using PERMANOVA.

Contrary to our expectations, OTU richness, Shannon diversity, phylogenetic diversity, and Pielou evenness were not significantly different between the fungal communities sampled in forest and vineyard, regardless of the amplicon used (Table 2). We did not find significant variation between vineyards for any diversity indices (Table 2). On the other hand, we found that habitats exhibited significantly different community structure regardless of the distance matrix employed: the ITS2 Bray-Curtis matrix (PERMANOVA: *F*_1,34_ = 6.94, *P* < 0.0001) or the D2 weighted-Unifrac matrix (PERMANOVA: *F*_1,34_ = 42.49, *P* < 0.0001). Indeed, we can visualize that fungal communities associated with forest and vineyard samples are clearly separated in the NMDS plot (Fig. 5).

**Table 2:** Fungal diversity indices associated with Carménére vineyards and sclerophyllous native trees. Results of linear mixed models for the diversity indices estimated from fungal communities sampled from vineyards and adjacent native trees. The *t* values, degrees of freedom and *P* values for habitat (fixed effect) are obtained from lmer summary. *P* values of vineyards (random effect) are based on likelihood-ratio tests (*χ*^2^).

**Diversity index**	**Amplicon**	**Habitat**	**Vineyard**
OTU richness	ITS2	*t*_10_ = − 0.56(*P* = 0.59)	${\chi }_{1}^{2}$= 0.18 (*P* = 0.67)
	D2	*t*_10_ = − 1.18(*P* = 0.27)	${\chi }_{1}^{2}$= 0.26 (*P* = 0.61)
Shannon diversity	ITS2	*t*_10_ = − 0.52 (*P* = 0.61)	${\chi }_{1}^{2}$= 0.07 (*P* = 0.80)
	D2	*t*_10_ = − 1.19 (*P* = 0.26)	${\chi }_{1}^{2}$= 0.07 (*P* = 0.79)
Phylogenetic diversity	D2	*t*_10_ = − 1.53 (*P* = 0.16)	${\chi }_{1}^{2}$= 0 (*P* = 1)
Pielou evenness	ITS2	*t*_10_ = − 0.35 (*P* = 0.73)	${\chi }_{1}^{2}$= 0.01 (*P* = 0.93)
	D2	*t*_10_ = − 1.38 (*P* = 0.20)	${\chi }_{1}^{2}$= 0.22 (*P* = 0.64)

## Discussion

### Effect of agriculture management and plant tissues on fungal communities

We found that vine leaves had higher fungal diversity and more unique OTUs than grape berries. On the other hand, the abundance of *D. tassiana* was extremely high in grape berries and vine leaves similar to findings reported by Bokulich et al. (2014), Piao et al. (2015), Pinto et al. (2015) and Taylor et al. (2014). We also found that diversity indices were significant higher in vine leaf than grape berry samples. It is possible that lower fungal diversity found in grape berries is due to sugar compounds found in the grape surface, which can constrain the number of fungal species capable of inhabiting this type of habitat. Indeed, we sampled mature grape berries, which have higher sugar concentrations than growing grape berries (Mane et al., 2017).

Conventional agriculture contributes to biodiversity loss, agrochemical pollution, and soil degradation and reduction of ecosystem services compared to organic farming systems (Reganold & Wachter, 2016). Moreover, several studies have shown that insecticides and fungicides have negative effects on biodiversity, and insecticides also reduce the efficacy of biological control in farming systems (Gabriel et al., 2010;  Geiger et al., 2010; Bevivino et al., 2014). In the present study, we found that diversity indices were similar between conventional and organic vineyards. Conversely, we found significant effects of agriculture management on fungal community structure regardless of the amplicon analyzed. Hartmann & Widmer (2006) report that the structure of soil microbial communities was more sensitive to the agricultural management than standard diversity estimations, similarly to our findings. In this context, our results do not agree with previous studies which have shown that fungal communities present in grapevines are not affected by biocontrol agents or synthetic fungicides (Kecskeméti, Berkelmann-Lohnertz & Reineke, 2016; Perazzolli et al., 2014). Indeed, our results suggest that fungal community structure associated with grapevine phyllosphere is sensitive to agricultural treatments. However, our experimental design was not planned to evaluate which of the different practices (i.e., fungicide application, tillage, etc.) is responsible for the patterns found in the present study. Therefore, manipulative experiments are needed to evaluate how single and/or combined agricultural practices influence these fungal communities.

According to taxa abundance, we did not found significantly different fungal species abundances between organic vineyards and conventional vineyards. Interestingly, besides *D. tassiana*, we found a high abundance of *A. pullulans* both in organic vineyards and forest samples. Previous studies have also found *A. pullulans* enriched in organically managed grapevines (Grube, Schmid & Berg, 2011; Martins et al., 2014). This increased abundance of *A. pullulans* in organic vineyards compared to conventional vineyards could be explained because this species is able to tolerate copper- and sulfur-based pesticides, which are permitted in organic management (Gadd & De Rome, 1988; Killham, Lindley & Wainwright, 1981). On the other hand, the application of Kresoxim-methyl in conventional vineyards has been shown to reduce *A. pullulans* abundance in Japanese pear leaves (Chung, Deepak & Ishii, 2013).

### Fungal communities in native forests and vineyards

We found a high proportion of shared fungal OTUs between vineyards and native forests (79.7%), indicating that most of the taxonomic units were present in both habitats and supporting the idea that “everything is everywhere, but the environment selects” (Baas Becking, 1934). Additionally, the diversity indices were similar between forests and vineyards. However, community structure was different between habitats, which may have been driven by differences in the composition and relative abundances of OTUs. Indeed, the most abundant OTUs in the vineyard samples included species as *D. tassiana*, *Didymella sp.* and *A. eichhorniae*. In contrast, *A. pullulans* and *E. populi* were the most abundant species in the forest samples. *D. tassiana* and *A. pullulans* have been previously reported as highly abundant in vineyards (Bokulich et al., 2014; Setati, Jacobson & Bauer, 2015), suggesting that these taxa represent the core microbiome of vineyards regardless of grape variety (Cabernet, Carménère, Chardonnay, Merlot, Zinfandel) and geographic region (Chile, Italy, South Africa, United States) (Bokulich et al., 2014; Pancher et al., 2012; Setati, Jacobson & Bauer, 2015).

In both forests and vineyards, we also found some OTUs identified as wine fermenting yeasts (e.g., *Hanseniaspora*, *Metschnikowia*, *Saccharomyces*), yet the relative abundance of these yeasts was very low in both habitats. This finding is consistent with previous studies showing that *S*. *cerevisiae* is rarely found on healthy grape berries (Barata, Malfeito-Ferreira & Loureiro, 2012) but is highly abundant in grape musts when alcoholic fermentation has begun (Bagheri, Bauer & Setati, 2015). However, it has been reported that native forests near vineyards are sources of fermenting yeasts. Specifically, fermenting yeasts have been found on *Nothofagus* (Southern beeches) in North Patagonia (Libkind et al., 2011) and New Zealand (Serjeant et al., 2008), and also on *Quercus* (oaks) in Portugal (Sampaio & Gonçalves, 2008). Furthermore, another study has also shown that the dispersal of foliar fungi is not locally limited; specifically, the composition of airborne fungal communities does not differ between vineyard and forest patches (Fort et al., 2016). Thus, it can be seen that forest-derived microorganisms can influence vineyard microbiota and vice versa. In a previous study, we have shown that the greater the geographic distance between vineyards, the more different is the fungal community structure associated with grape berries (Miura et al., 2017). This finding implies that local- or landscape-scale factors such as geographical barriers and variation in ecological niches could affect the spatial patterns of the microbial communities of the grapevine phyllosphere. This being said, further studies are needed to quantify the role of native forests on vineyard microbiota.

Regarding microbial diversity, host taxonomic identity has been shown to be an important driver of phyllosphere microbial community composition (Kembel & Mueller, 2014; Whipps et al., 2008) Thus, different plant communities should have different abundances of microbes and their microbial communities should differ among them. However, we found that both habitats had similar levels of diversity despite that forest samples were collected from multiple plant species. This result differs with our hypothesis and with another study showing higher fungal OTU richness on forest leaves compared to vine leaves (Fort et al., 2016). Additionally, we found that forests and vineyards share 80% of OTUs found in this agroecosystem. A possible explanation for this could be due to the unique characteristics of sclerophyllous trees which have leaves with thick and hard cuticles that can act as a barrier for invasive microorganisms (Bringel & Couée, 2015; Yeats & Rose, 2013). Also, these species produce aromatic volatile compounds with antimicrobial effects (Velásquez & Montenegro, 2017). Nevertheless, the phyllosphere community structure was significantly different between vineyards and native forests. These differences were mainly due to differences in the abundance of some OTUs. For example, the relative abundance of *E. populi*, a pathogen species that causes powdery mildew ( Kassemeyer & Berkelmann-Löhnertz, 2009), was higher in vineyard compared to in forest samples. In contrast, OTUs identified as *A. pullulans* were more abundant in forest than in vineyard samples. This species has been shown to be capable of copper-detoxification (Gadd & De Rome, 1988) and is also a biocontrol agent for grapevine diseases (Compant & Mathieu, 2016).

## Conclusion

Interestingly, even though the forest samples were collected from multiple plant species, the fungal diversity of these native sclerophyllous tree leaves was similar to that of vine leaves. Nonetheless, community structure differed greatly between native forests and vineyards. We argue that the characteristics of sclerophyllous tree leaves might pose strong selective pressure on fungal assemblages, and this could be an important factor influencing the landscape-specific diversity of phyllosphere fungal communities in Chilean Mediterranean ecosystems.

Interestingly, our results also demonstrate that agricultural practices do not affect the fungal diversity but they do affect the community structure of the fungal communities associated with grapevines. On the other hand, the similarity of vineyard- and forest-associated fungal communities suggests that native forests could greatly contribute to vineyard phyllosphere microbial communities. These studies are clearly worthy of further investigation to determine the extent to which these natural ecosystems act as reservoirs of microbial diversity. Overall, this study argues that a better understanding of the relationship between native forests and agroecosystems is needed for maintaining and enhancing ecosystem services provided by natural ecosystems. Specifically, knowledge about fungal diversity can be used to identify fermenting yeasts valuable for the wine industry, and furthermore this information can be used to better evaluate the ecosystem services provided by this native ecosystem.

##  Supplemental Information

10.7717/peerj.5715/supp-1 Figure S1 Rarefaction plots for ITS2 and D2 fungal amplicons Rarefaction plots indicating the number of OTUs for (A) ITS2 and (B) D2. Panel C shows the relationship between phylogenetic diversity and the number of D2 reads. Samples were collected from forest leaves (dark green), vine leaves (green), and grape berries (Purple).

10.7717/peerj.5715/supp-2 Table S1 Comparison of relative abundances of operational taxonomic units (OTUs) between conventional and organic vineyards This table indicates the OTU identity, the normalized mean between groups, the log_2_ fold change and its standard error, the Wald statistics and their *P*-values, the *P*-value corrected for multiple comparisons (False Discovery Rare; FDR), and the taxonomic assignment from phylum to species. Red rows indicate OTUs that were significantly more abundant in conventional vineyards and blue rows indicate OTUs that were significantly more abundant in organic vineyards.

10.7717/peerj.5715/supp-3 Table S2 Comparison of relative abundances of species between conventional and organic vineyards using ANOVA This table indicates the taxonomic assignment for each OTU, the F statistic and its *P*-values, the *P*-value corrected for multiple comparisons (False Discovery Rare; FDR), and the mean relative abundance of each species in the vineyards and forest. No species showed significantly different abundance between vineyards with different agricultural management.

10.7717/peerj.5715/supp-4 Table S3 Comparison of relative abundances of operational taxonomic units (OTUs) between grape berries and vine leaves This table indicates the OTU identity, the normalized mean between groups, the log_2_ fold change and its standard error, the Wald statistics and their *P*-values, the *P*-value corrected for multiple comparisons (False Discovery Rare; FDR), and the taxonomic assignment from phylum to species. Green rows indicate OTUs that were significantly more abundant in vine leaves and purple rows indicate OTUs that were significantly more abundant in grape berries.

10.7717/peerj.5715/supp-5 Table S4 Comparison of relative abundances of species between grape berries and vine leaves using ANOVA This table indicates the taxonomic assignment of each OTU, the F statistic, and its *P*-values, the *P*-value corrected for multiple comparisons (False Discovery Rare; FDR), and the mean relative abundance of each genus in vineyards and forest. Green rows indicate species that were significantly more abundant in vine leaves and purple rows indicate species that were significantly more abundant in grape berries.

10.7717/peerj.5715/supp-6 Table S5 Comparison of relative abundances of species between vine and native forest leaves using ANOVA This table indicates the taxonomic assignment of each OTU, the F statistic and its *P*-values, the *P*-value corrected for multiple comparisons (False Discovery Rare; FDR), and the mean relative abundance of each species in the vineyards and forest. Green rows indicate species that were significantly more abundant in native forests and purple rows indicate species that were significantly more abundant in the vineyards.

10.7717/peerj.5715/supp-7 Table S6 Comparison of relative abundances of operational taxonomic units (OTUs) between vine leaves and native forest leaves This table indicates the OTU identity, the normalized mean between groups, the log_2_ fold change and its standard error, the Wald statistics and their *P*-values, the *P*-value corrected for multiple comparisons (False Discovery Rare; FDR), and the taxonomic assignment from phylum to species. Green rows indicate OTUs that were significantly more abundant in the native forests and purple rows indicate OTUs that were significantly more abundant in vineyards.

## Additional Information and Declarations

### Data Availability

The following information was supplied regarding data availability: FASTA, BIOM, metadata and OTU table files are deposited at Figshare:
https://figshare.com/articles/Raw_data_fungi_manuscript/6222473. Sequences are also available in GenBank under BioProject number PRJNA392462.
