# Supplementary figures and images for: Effects of agricultural management on phyllosphere fungal diversity in vineyards and the association with adjacent native forests
Source: PeerJ. 2018 Oct 29;6:e5715. doi: 10.7717/peerj.5715 (PMC6211267; doi:10.7717/peerj.5715)

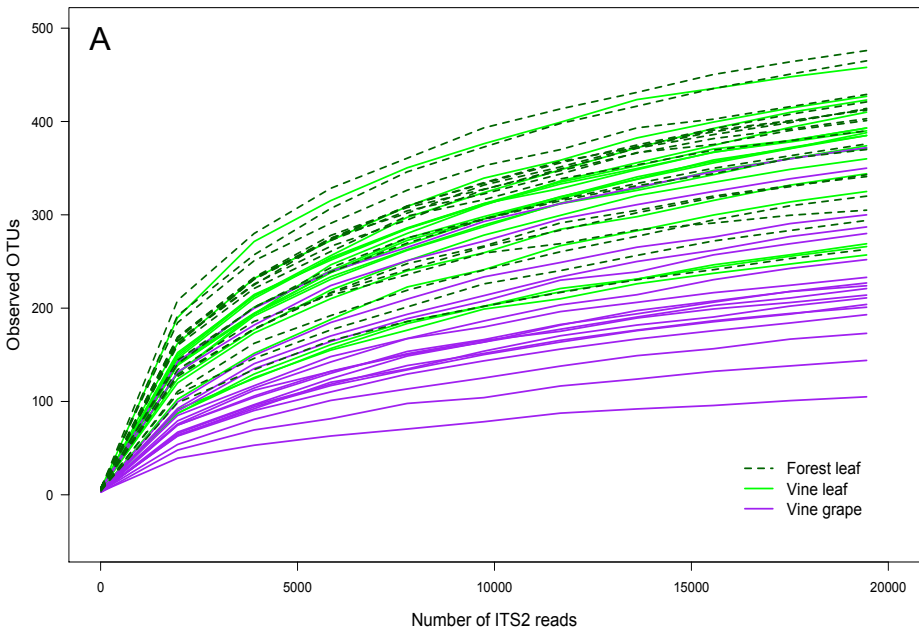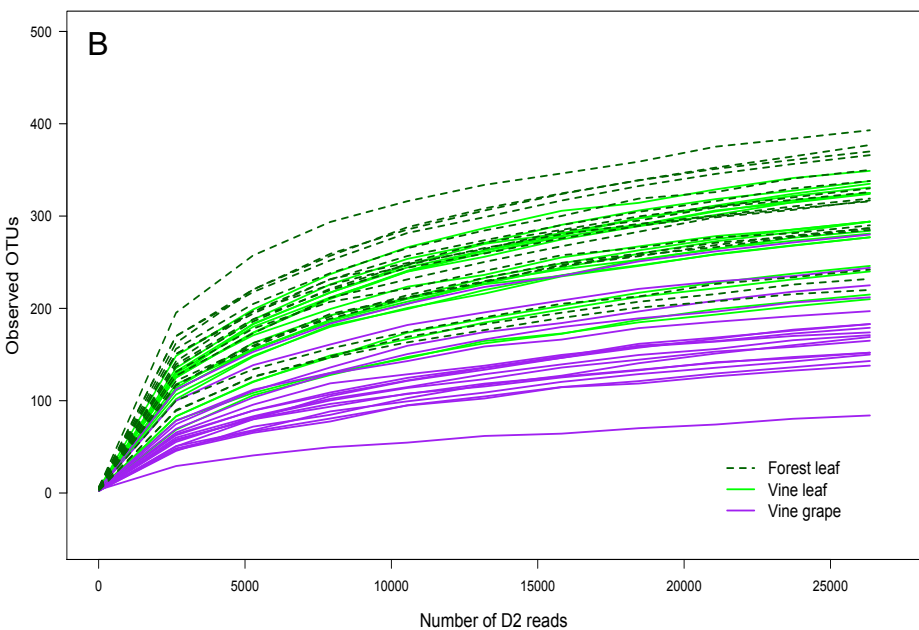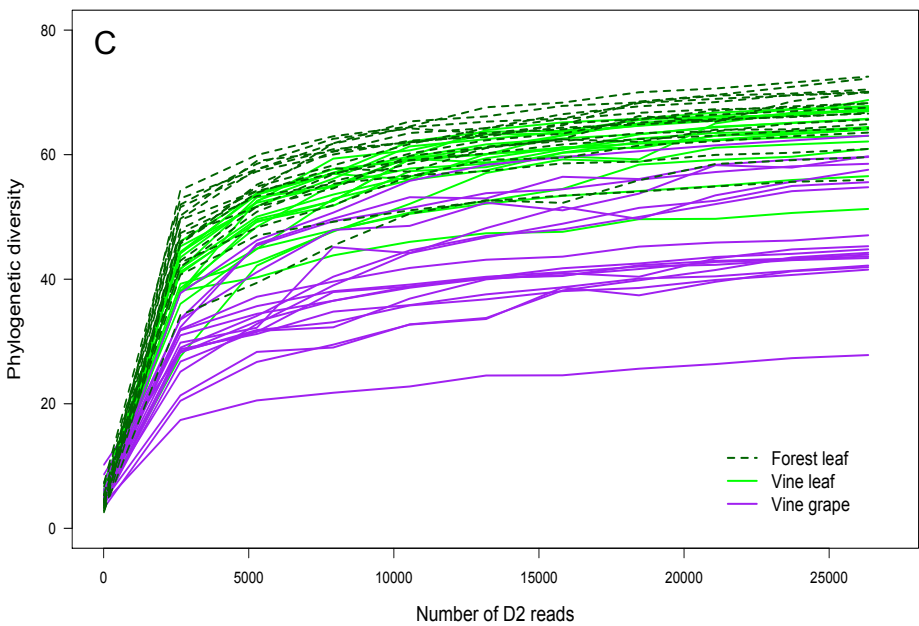

Supplement: Figure S1 — Rarefaction plots indicating the number of OTUs for (A) ITS2 and (B) D2. Panel C shows the relationship between phylogenetic diversity and the number of D2 reads. Samples were collected from forest leaves (dark green), vine leaves (green), and grape berries (Purple). [file peerj-06-5715-s001.pdf]
